# Reducing Sexual and Reproductive Health Inequities Between Natives and Migrants: A Delphi Consensus for Sustainable Cross-Cultural Healthcare Pathways

**DOI:** 10.3389/fpubh.2021.656454

**Published:** 2021-05-13

**Authors:** Pedro Candeias, Violeta Alarcão, Miodraga Stefanovska-Petkovska, Osvaldo Santos, Ana Virgolino, Sónia Pintassilgo, Patrícia M. Pascoal, Andreia Silva Costa, Fernando Luís Machado

**Affiliations:** ^1^Instituto de Saúde Ambiental, Faculdade de Medicina, Universidade de Lisboa, Lisboa, Portugal; ^2^Centro de Investigação e Estudos de Sociologia, ISCTE - Instituto Universitário de Lisboa (ISCTE-IUL), Lisboa, Portugal; ^3^Unbreakable Idea Research, Painho, Portugal; ^4^Centro de Investigação em Ciência Psicológica, Faculdade de Psicologia, Universidade de Lisboa, Lisboa, Portugal; ^5^Digital Human-Environment Interaction Lab, Universidade Lusófona, Lisboa, Portugal; ^6^Centro de Investigação, Inovação e Desenvolvimento em Enfermagem de Lisboa, Escola Superior de Enfermagem de Lisboa, Lisboa, Portugal

**Keywords:** sexual and reproductive health, health equity, migrants, Delphi panels, inequities and inequalities in health

## Abstract

The increasing number of international migrants (ranging from 153 million in 1990 to ~272 million in 2019) brought to attention the wide variation of national contexts concerning the policy measures to protect migrants' rights and ensuring their equal access to basic and essential services, namely in health. Sexual and Reproductive Health (SRH) is a key component to the overall health and quality of life and is impacted by power inequities inherent to society's institutions, environment, economics, and culture. In Portugal, guidelines for intervention in SRH are insufficient, a gap that is more pronounced with migrant populations due to the absence of culturally sensitive indicators to assess and monitor SRH. The aim of this work was 2-fold: to identify good practices in the SRH field, with a particular focus, whenever possible, on migrant populations, and to identify relevant and inclusive indicators to monitor SRH in Portugal. A Delphi panel (via online survey) with 66 experts (researchers, teachers, and health professionals) and 16 stakeholders (non-governmental organizations, civil society, and governmental organizations) was implemented in two rounds. Panelists were asked to state their level of agreement (5-point Likert-type scale) regarding four different SRH areas: Sexual Health, Reproductive Health, Social-Structural Factors, and Good Practices. Items were based on literature review and a World Café with 15 experts and stakeholders. Participation rate was 68% and response rate was 97% on the first round. From the initial list of 142 items, a total of 118 (83%) items were approved by consensus. Findings may provide extended opportunities for the healthcare system to engage in better informed decisions and more inclusive and integrative strategies regarding SRH, contributing to build political measures toward sexual and reproductive justice.

## Introduction

According to the World Migration Report 2020, the total number of international migrants is estimated to be almost 272 million, with nearly two-thirds being labor migrants and nearly half being female (1). In 2020, female migrants accounted for 47.6% of all migrants in high-income countries, 48.2% in middle-income countries, and 50.9% in low-income countries. The share of female migrants was highest in North America (51.8%) and Europe (51.4%). In addition, the current estimated number and proportion of international migrants already surpasses the projections made for the year 2050 (1).

**Figure 1:**
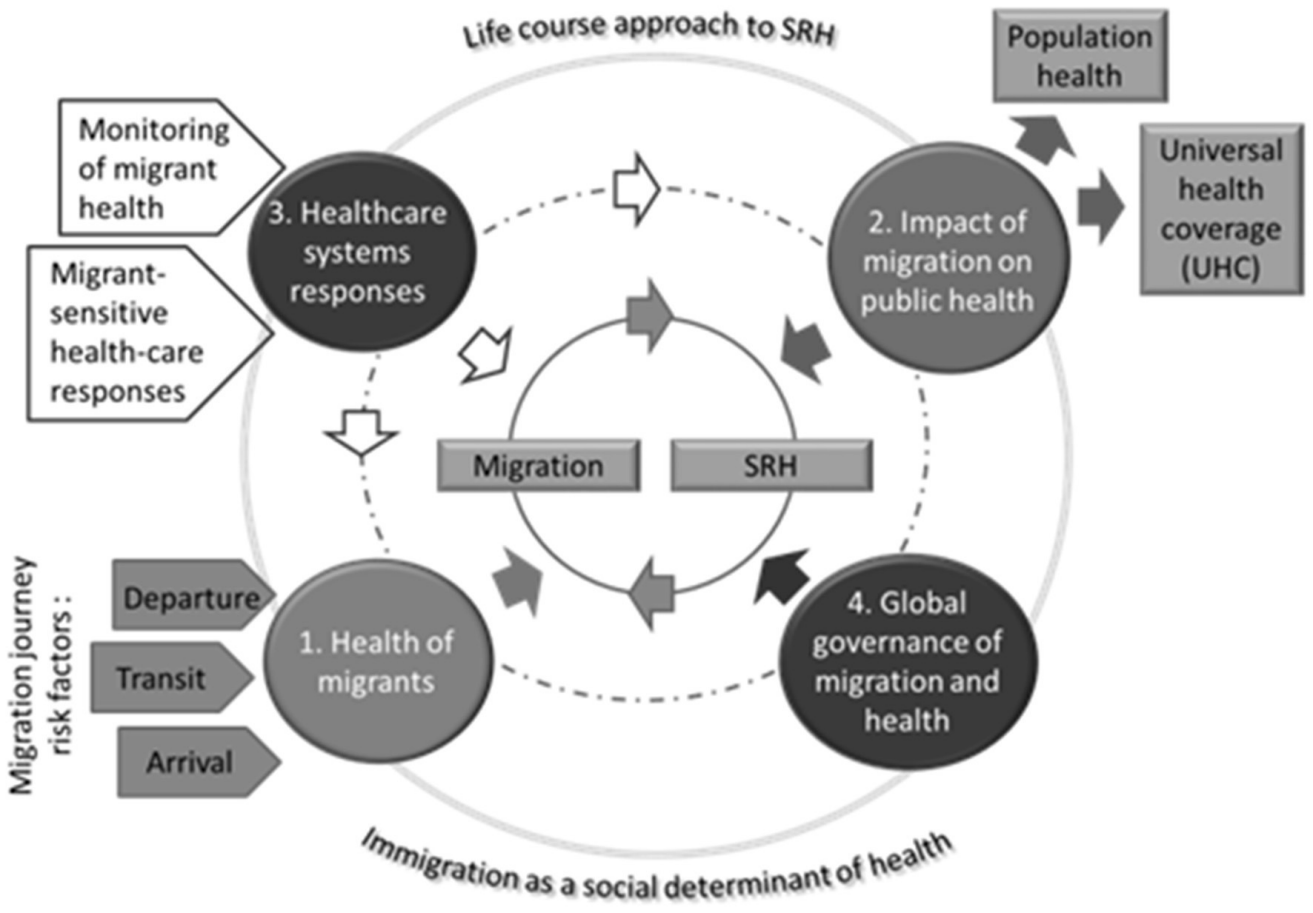
Key aspects and determinants of migration and health. Source: Authors own compilation based on (1, 2).

The relationship between migration and health is well-established in the literature. In general, the existing research studies investigate and suggest interventions in four key aspects of the relationship between migration and health: (1) health of migrants; (2) the impact of migration on public health; (3) the response of the healthcare system; and (4) the global governance of migration and health (1, 2). Each of these aspects is discussed in detail bellow and illustrated in Figure 1.

1. Health of migrants—The area that concerns the health of migrants focuses on the differences in the health status between the migrants and their counterparts in the origin and destination country. The determinants of changes in the health status of the migrants are dependent on the exposure to risk factors at departure, during transit and at arrival (3–5). In example, some migrants are faced with increased risk for sexual violence and exploitation during the migration journey (5).
2. Impact of migration on public health—The second key element is public health and the global target of universal health coverage (UHC) (6). Access to affordable quality and culturally competent healthcare is an important concern for all vulnerable groups, especially migrant workers, and poses a neglected challenge to progress toward universal health coverage. Therefore, national systems should identify migrant population in order to understand the scale of migration, develop evidence-based policies, and know the extent to which refugees and labor migrants are able to access health and other social services. As 64% of all migration is related to work, it would therefore benefits the host country to invest in their health (7).
3. The healthcare systems response is one of the essential elements of the intersection between migrants and health. Developing systems that are sensitive to migrants cultural and health characteristics would result with multiple positive consequences for the health of the migrants, their families and the communities in which they live. In example, services for sexual and reproductive health are typically under-utilized by migrant and refugee communities and certain studies indicate a lower utilization rate of health services of migrant, compared to native women (8) due to lack of knowledge about available services and how to access them, language barriers, differences in the cultural understanding of health, healthcare and health-seeking behavior, inability of the healthcare system and workforce to identify and understand the specific needs and circumstances of the migrant population, as well as unresolved administrative status of the person (9).
4. Finally, the global governance of migration and health encompasses the integration of equity, accountability, impartiality, fairness, justice and probity into the global governance processes (10).

During 2019, the stock of foreigners in Portugal accounted for 590,348 people (5.7% of the total population) (11). In terms of age, 57.5% of foreigners were aged between 15 and 44 years with a 50/50 ratio between men and women (12). The Portuguese Observatory for Migration publishes an annual statistical report of the migrant integration indicators that allows access to organized indicators on social, economic, educational and civic indicators, based on nationality. It also provides an understanding of the challenges that persist in monitoring the integration of migrants in Portugal, namely in the health dimension, with indicators related to access to and use of health services, and the needs of resident populations and health systems (13). As in other countries, in Portugal there are differences in health indicator outcomes between migrants and the autochthonous population placing migrants in an unfavorable position in terms of their access and utilization of healthcare services, specifically concerning certain health risk factors such as inadequate diet, tobacco, and alcohol consumption (5). The systematic health status differences between natives and migrants may reflect inequities in the accessibility of health services, as well as diverse health inequalities and health protection needs due to the socio-economic characteristics of the population (13–16). By definition inequity refers to unfair, avoidable differences arising from poor governance, corruption or cultural exclusion while inequality simply refers to the uneven distribution of health or health resources as a result of genetic or other factors or the lack of resources (17). Inequality was listed as a global risks in 2012, while in 2017 it was considered that in the following decade the rising income and wealth disparity will be one of most powerful determinants of global development (18). An example to the significance of addressing this issue can be found within the issue of maternal deaths in low- and middle- income countries. Although the majority of maternal deaths are avoidable through quality obstetric care, such as cesarean section, evidence suggests inequality and inequities among women in low-and middle-income countries concerning obstetric services. Findings from a 19 year study in Tanzania indicated that women who were uneducated, poorest/poor, living in rural settings and from certain regions demonstrated lower utilization of obstetric services (19). In regards to Portugal, the country has already implemented the concrete measures in the past 5 years to increase women's access to comprehensive sexual and reproductive health services, regardless of marital status and age as well as support for family planning and specific programs to ensure the access of adolescents and youth to sexual and reproductive health information (20). However, intergroup differences are observed between migrant and domestic population. A study found that the families of newborn children in Amadora and Sintra Council districts (districts with the highest proportion of migrants) face increased socio-material deprivation compared to the general population of the Greater Metropolitan Area of Lisbon. Their health vulnerability is reflected in the greater fetal and post-natal mortalities and more deaths during pregnancy, mainly due to infectious diseases (21). Another study that used data on births registered between 1995 and 2002 and classified by reported nationality of mothers, found that among African births there was an increase in births to teenaged mothers and a decline to mothers from advantaged socioeconomic backgrounds. Additionally, in the investigated period there was a decline on mean birth weight among African babies that was found to be associated with socioeconomic advantage (22). The impact of structural inequities and socioeconomic health determinants in ethnic and migrant health inequities has increased during the COVID-19 pandemic (23). In this context, it is essential to address the wide variation of national experiences in what concerns policy measures to protect migrants' rights and well-being and ensuring equal access to essential services, with special emphasis on healthcare.

### Addressing Sexual and Reproductive Health Related Inequities

Sexual and reproductive health (SRH) is shortly defined by the World Health Organization (WHO) (24) as a state of physical, emotional, mental and social well-being related to sexuality. The health issues covered by SRH include, but are not limited to improving maternal and newborn care, providing high quality services for family planning, eliminating unsafe abortion, combatting sexually transmitted infections, and promoting sexual health, which includes protecting sexual rights, improve sexual function and promote sexual pleasure free of coercion (25). Although the foundation of SRH health outcomes lies in individual behavior, there is an array of forces and systems shaping the conditions of migrants' daily lives that cannot be ignored (26). Therefore, effective SRH can only be achieved when considering the full range of factors that make a critical difference to health outcomes. This is especially important since services for SRH are typically under-utilized by migrant and refugee communities, when compared to the native population (8). Reasons include lack of knowledge about available services and how to access them, language barriers, differences in the cultural understanding of health, healthcare and health-seeking behavior, inability of the healthcare system and workforce to identify and understand the specific needs and circumstances of the migrant population, as well as unresolved administrative status of the person (9).

One of the novel approaches in sexual health monitoring and evaluation with specific focus on migrant population highlights the importance of envisaging the diversity of individual needs at various points across life course and in various settings or circumstances (27).

**Figure 2:**
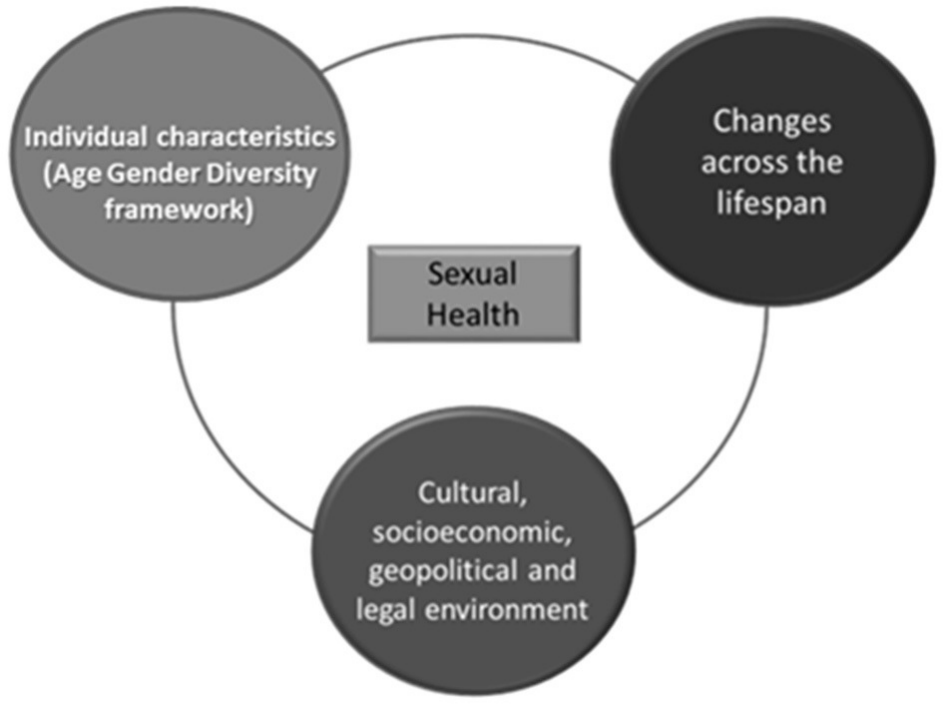
Determinants of sexual health. Source: Authors own compilation based on WHO operation framework of SRH interventions (10) and the United Nations Refugee Agency's (UNHCR) Age, Gender, and Diversity (AGD) framework (11).

The importance of this interaction is highlighted in the fifth key principle from the WHO operation framework—*Diversity of needs across life course and populations* (28, 29). This principle highlights three forces that shape SRH—individual, environment and time. More specifically, it views sexual health as a complex interaction between individual characteristics, the role of the cultural, socioeconomic, geopolitical and legal environment in SRH outcomes, but also the changes incurred over time and across the lifespan. In addition this goal is complementary with the UN's Sustainable Development Goal 3 (SDG 3) for 2030 which aims to “ensure healthy lives and promote well-being for all at all ages.” This goal crosscuts with the other SDGs (30, 31). Hence, in regards to SRH the intersectional approach envisages the importance of ‘the different stages in one's life cycle' and of being aware of where people are in the life cycle as their capacities and needs change over time. It has also been incorporated into the United Nations Refugee Agency's (UNHCR) Age, Gender, and Diversity (AGD) framework (32) (Figure 2). This framework sets out a definition of diversity for ‘one community, many people', and draws attention to the roles and needs of women and girls, men and boys, children (including adolescents), people who are lesbian, gay, bisexual, transsexual, or intersex (LGBTI), older men and women, disabled people, and those belonging to national or ethnic, religious and linguistic minorities or indigenous groups (33). In accordance with the AGD framework, the approach should be used to plan, program, implement, monitor, and evaluate the relevant indicators. The adoption of the life-course approach promotes functional ability of the individual, as the sum of the individual and environmental attributes that enable a person to be or do what they have reason to value, that in turn enables well-being and is interdependent with the realization of rights (31, 34). Estimates suggest that long-term investment in the life-course approach can results in with benefits that are not limited to health, but extend to social and economic development as well (31). In example, the reduction of preventable diseases in low- and middle- income countries has resulted in their increased economic growth (35). An example of a more locally oriented action is the Madsen's Institute for Tribal and Rural Advancement program that utilized the life-course approach in their cross-sectorial programs to transform the health of people in 48 villages in Orissa in India by targeting primarily malaria control and afterwards including other interdependent health, educational, environmental and poverty-reduction goals. The result was a halved infant mortality rate over a 15 year period, and a range of advances in the areas of health, social and developmental areas, that in contrast remained very low in villages not covered by the program (36).

This fifth key principle is complemented by two main approaches found in the literature: *the life course approach to SRH* (37) and *the migration as a social determinant of health* (38). The first approach argues that events at different stages of life must be understood as fundamentally connected (39). According to this approach individual life courses are composed of multiple, simultaneously occurring trajectories through various dimensions of life (e.g., family, work, sexuality). Each trajectory extends from birth until death and can be divided into a sequence of transitions (i.e., retirement or virginity loss). This framework posits that sexual beliefs and behaviors result from individuals' lifelong accumulations of advantageous and disadvantageous experiences—social, psychological, and physiological—and their adoption or rejection of sexual scripts within specific socio-historical contexts.

In regards to migration, in their lives migrants undergo experiences that ultimately affect their health in a setting characterized with legal, cultural, social, economic, and behavioral barriers. Migration itself can be a strong determinant of physical and mental health. Therefore, it should be viewed as a social determinants of health which emphasizes the *racialized-gendered social determinant of health*—the dominance of race and gender identities, along with other identities such as social class, sexual orientation, age, ethnicity and nativity, and legal status, that form the basis for education and health frameworks (40). It is considered that the ability to treat migration as a social determinants of health has the potential to result in a comprehensive and targeted response to the health of the populations affected by the global phenomenon of migration (38).

Taking into consideration all the relevant aspects, approaches and arguments that surround SRH and migration, the Delphi method was chosen to identify guidelines for intervention with migrant populations in Portugal that are currently insufficient due to the absence of culturally sensitive indicators to assess and monitor SRH. The Delphi method has been commonly applied in the selection processes of health indicators where group opinion is needed from an audience with varied views, such as in the health field (41). This method has been used in studies to select indicators on healthcare services (42, 43), perinatal health in Europe (44, 45), health inequalities and inequities (46, 47) and population health (41). Therefore, this Delphi study was implemented to generate consensus on:

- what constitutes good practices in the SRH field, with emphasis on SRH equity across migrant populations;
- relevant and inclusive indicators to monitor SRH, namely among migrants, in Portugal.

## Materials and Methods

The study was approved by the Ethics Committee of the Centro Académico de Medicina de Lisboa (CAML). A Delphi panel approach was used to achieve agreement on the best indicators to monitor SRH in Portugal, establishing good practices in the SRH field to both the host and the migrant populations. In general terms, the Delphi method assumes that the opinion of experts can have a scientific application (48). It consists of a participatory methodology that aims to generate consensus, where several experts participate, building consensus between their ideas on the subject in question, but without direct confrontation of opinions (49, 50). To this end, it implies a series of anonymous questionnaires with the particularity of the respondents having access to the group's statistics (48). This method has been used both in the field of social policies and public health (51). It has the potential to obtain viable data that allow informing policy makers (48). The obtained results are based, to a large extent, on personal perspectives, drawing on the experiences and knowledge of the group of qualified specialists carefully selected, with a multidisciplinary vision that allows the establishment of objectives and interventions (52).

### Preliminary List of Indicators

**Figure 3:**
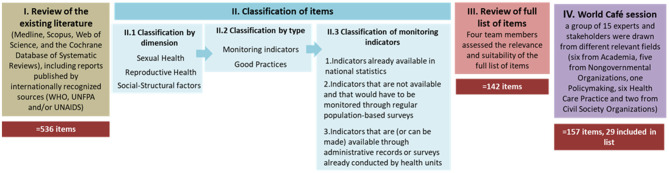
Development of the Delphi form.

The development of the Delphi form is illustrated in Figure 3. The set of items/indicators included in the Delphi panel were based on two distinct but still complementary approaches: a literature review and an initial input using expert opinion, collected through the World Café method. Firstly, a review of the existing literature that covered several sources that have been reflecting on issues related to sexual and reproductive health and rights and migration (Medline, Scopus, Web of Science, and the Cochrane Database of Systematic Reviews), including reports published by internationally recognized sources (WHO, UNFPA, and/or UNAIDS), was implemented to better understand the scenario of the current needs and gaps in existing data, and the nature of indicators that the project should entail (29, 41, 53–64). This collection of items resulted in a first list of 536 entries, which were divided into monitoring indicators (447 items) and good practices (88 items). Monitoring indicators are understood as standardized measures, which allow measuring processes that change over time and are considered essential for the creation of health policies (65). Good practices can be defined as an action, which can be compared with an alternative action and where can be established a link between this action and some desirable outcome (66). In other words, the good practices concern measures that must be taken, indicators concern ways of quantifying the impact of the measures and/or helping to define better or more appropriate measures.

For the presentation on the questionnaire forms, and in the subsequent analyses, the identified indicators were classified into three dimensions in accordance to the WHO operational definition of SRH (10): (1) Sexual Health, (2) Reproductive Health and (3) Social-Structural Factors. The indicators were finally subjected to segmentation between: (a) indicators already available in national statistics; (b) indicators that are not available and that should be monitored through regular population-based surveys; and (c) indicators that are (or can be made) available through administrative records or surveys conducted by healthcare units.

Secondly, complementary to this literature-based collection of items, a World Café (WC) (67) session was undertaken to enable obtaining new indicators, potentially different from those obtained through the literature review and more adapted to the national context. The WC method can be defined as a structured conversational process, that facilitates a group discussion, focused on a specific theme and that allows the construction of “collective wisdom” (68). The WC encourages people to speak in a relaxed environmental context. It is based on the assumption that *cafes* provide a creative atmosphere (69). Another assumption is that small group organization facilitates learning, in addition to being less intimidating, and allows everyone the opportunity to express themselves and comment others' ideas (70). For this purpose, a group of 15 experts and stakeholders were drawn from different relevant fields (Academia, Non-governmental Organizations [NGOs], Policymaking, from Healthcare Practice and Civil Society Organizations; multiple affiliation was possible). These specialists were selected due to their experience and expertise in the field and were asked to think about SRH indicators, in general, and specific SRH indicators suitable for migrant populations while considering the three dimensions of WHO (Sexual Health, Reproductive Health, and Social-Structural factors). Items listed by the specialists, together with the ones from the initial set were included in the final list of items launched for discussion within the Delphi panel.

The full list of items was then reviewed by four members of the research team who assessed the items' relevance and suitability. The following five criteria were used in the review of the items: (1) *Repetition or equivalence*. In situations of equal or quite similar indicators, the one that was formulated more clearly was chosen. (2) *Adequate clarity/depth*. Items that were not too abstract / vague / general were privileged. On the other hand, items that were too specific were avoided, as they could be outside the domain of some of the experts. As example “Time frame and coverage of national policy on abortion and fetal sex determination.” This item mixes time frame with coverage, therefore being unclear. (3) *Link to the theme*. Items more directly close to the SRH intervention areas were privileged. As example: “Percentage of people protected against catastrophic/impoverishing out of pocket health expenditure.” It falls out of the SRH scope. (4) *Feasibility*. Since the outcome indicators are indicators derived from statistics or administrative records, it was accounted whether the indicator could be measured. As example “Percentage of facilities that report not experiencing a stock-out of a modern form of contraception in the past 6 months.” This item was not included as it would imply monitoring the inventories of all facilities at the national level. Note that no distinction was made between existing indicators and indicators that would need to be created. (5) *Unidirectionality*. Since the objective of the outcome indicators is to be collected over time, in order to understand the effectiveness of the implemented measures, only unidirectional indicators were chosen. As example “Percentage of people who have had more than one sexual partner in the past 12 months.” In a public health frame, a greater number of sexual partners can imply a greater risk of chronic diseases (71). However, the freedom of choice in matters relating to own sexual life is a sexual right.

Each item was evaluated by two investigators who were unaware of their peers' endorsement (blind process). In case of doubt or disagreement, the item was discussed by the extended team of four members. A list of 142 items was reached to be discussed with the Delphi panel.

### Delphi Panel Recruitment and Formation

In order to tap on an adequate range of perspectives regarding SRH and migrants, a comprehensive list of experts and stakeholders with knowledge and experience in SRH among the migrant population living in Portugal was created. No quota criteria were used in relation to gender or geographic area of intervention, although recruitment has been the most inclusive as possible. In order to obtain an exhaustive list of participants, a web search was carried out on institutional sites of NGOs, civil society organizations, scientific societies, research, and teaching institutions. Research team members attended scientific events (congresses, seminars and workshops) in order to be able to establish personal contact with potential participants that had not been previously identified. After initial contacts were made, additional participants were included through a snowball referral. Snowball sampling, also known as “chain-referral-sampling” is a convenience sampling method (therefore not probabilistic) in which some of the participants recruit new participants through their network of contacts (72, 73). Attempts were made to distribute these sectors as evenly as possible. The following rationale was used to select members of each sector:

1. *Academia*—only demography, birth and related specialties were directly considered. In the case of migrations scholars, they were only considered if they were linked to the previously indicated specialties, or to migrations and health, i.e., migrations and demography, migrations and birth, migrations and health, etc. The specialties of family, sexuality and gender identity, gender violence or gender equality were not considered relevant to the case, unless they had some relation to the themes of intersectionality or migration.
2. *Civil society*—namely experts from migrant associations. Only those that acted on sexual, reproductive, intersectional, gender, and sexuality were considered. Recreational, legal rights, and support for young people associations were not considered.
3. *Non-governmental organizations*—only those having a professional activity related to migrations and health and to SRH were considered. For example, for the promotion of sexual and reproductive health and rights, or for the human rights of women in childbirth. Gender equality actors were not considered.
4. *Healthcare services or organizations*—comprised professionals from three sub-areas: (a) Public health, if they were specialized in working with migrant populations; (b) Gynecologists, obstetricians, and urologists; and (c) Sexologists, except for specialists in childhood sexology.
5. *Governmental organization*—included members of the central and local administration, and members of public institutes that had some connection to the issues under analysis.

Out of the 137 potential participants that had been initially identified, it was not possible to obtain a response from 28, either because they did not answer to the formal invitation sent by email, or because the email has bounced back. Furthermore, sixteen people were excluded because they replied to not having enough knowledge about the topic (though filling in the inclusion criteria). Only the responses of those 82 participants who fully completed the form (from 93 participants who accepted to participate) were used for this analysis.

### Development of Delphi Questionnaire

The Delphi was designed using Limesurvey^®^ online survey system. An invitation to participate was sent with the survey link to the questionnaire with a personalized access code, thus ensuring data confidentiality between experts' answers. Along with the questionnaire forms, all participants received an online consent form informing them on the project aims and their rights. The form was made up of six sections:(1) Introduction to the study and informed consent, (2) Socio-demographic characteristics, (3) Monitoring indicators of Sexual Health in Portugal, (4) Monitoring indicators of Reproductive Health in Portugal, (5) Monitoring indicators of social-structural factors with an impact on Sexual and Reproductive Health, and (6) Evaluation of good practices in Sexual and Reproductive Health. With reference to the indicators, sections Results, Discussion, and Conclusion, included information on how the indicator would be collected (by surveys of the population, through administrative data or through official statistics). Initially, three rounds of Delphi were planned (74). However, in agreement with certain literature that argues that it is possible to finish the panel at the end of the second round in case a satisfactory consensus is reached (75), the high consensus observed at the end of the second round determined that an additional third round was not needed. For each round, the opinion of the panelists about the suitability and relevance of each item were collected using a five-point Likert scale. The formulation of the questions and the answer options were the same in both rounds. In the sections that concerned the indicators, the replies were collected through the following item: “In your opinion, what is the relevance of each of the following indicators for the evaluation / monitoring of Sexual Health in Portugal?” and were recorded on a five-point Likert-type scale (*1* = *Totally irrelevant, 2* = *Irrelevant, 3* = *More or less relevant, 4* = *Relevant, 5* = *Totally relevant*). In the section concerning good practices, replies were collected through the following item: “In your opinion, to what extent do you agree with the fact that each of the following items is good practice in the field of Sexual and Reproductive Health in Portugal?” and were recorded on a five-point Likert-type scale (: 1 = “*I strongly disagree”*, 2 = “*I disagree”* 3 = “*I neither agree nor disagree,”* 4 = “*I agree,”* 5 = “*I strongly agree”)* (41, 76). Additionally, both sections included a “*no opinion/don't know how to answer*” option. Furthermore, an open question was included at the end of each section of the Delphi form, asking participants to propose new indicators or to suggest potential changes to the already included indicators.

Round 1 took place between 18 February 2020 and 5 March. Round 2 took place between 12 March 2020 and 31 March 2020. At the end of each round, the participants were presented with the anonymous aggregation of the results regarding the items approved and rejected. In the second round, participants had access to aggregated responses in items where no consensus had been reached with the aim to question the relevance of the indicators and their agreement with good practices. To reduce the dropout rates and the effect of non-response bias, personal reminders without inclusion in BCC (Blind Carbon Copy), were sent to the participants who did not complete the survey within the specified time and deadlines were extended.

### Data Analyses

Regarding group agreement rules, in Round 1, the same criterion was followed as that used in Freitas et al. (41) where the approval and rejection decision were based on the following criteria: 50% of “4” or “5” and at the same time no more than 1/3 (33.3%) of “1” or “2” would be accepted. Items with more than 50% of “1” or “2” would be rejected. In Round 2, the criterion was more demanding and based on literature that suggested as a criterion values between 60 and 90% according to what the researchers considered meaningful (77), in this way, only items that had more than 75% of “4” or “5” responses were approved. Items with lower approval percentages would be rejected.

In order to explore the obtained results, approval rates were used as a measure of consensus (78) and no opinion rates were calculated (79). The level of consensus among the panelists was assessed through the coefficient of variation (mean/standard-deviation) (80). The cut-off referred for a good degree of consensus was between 0 and 0.5 (81). This analysis was complemented with Kernel Density curves as a complementary method for analyzing panelists' consensus (41). Mean values were calculated by dimension and by round.

In order to analyze the changes of opinion by panelists between Round 1 and 2, the McNemar Test was used (82, 83). This test is similar to the chi-square test, but applicable to paired samples and dichotomous variables (2X2). It allows perceiving the change *vis-à-vis* stability of the panelists' position. The null hypothesis is that the respondents 'opinion does not change between R1 and R2 and the alternative hypothesis that the respondents' opinion changes between R1 and R2, either for greater acceptance or for greater rejection. A *p* < 0.05 was considered statistically significant.

Data analyses were done using Microsoft Excel 2011 and SPSS versions 23.

## Results

### Panel Participation

Of the 137 initial contacts that were selected to participate in the Delphi process, 93 were considered eligible (68%). The remaining 16 were excluded for the following reasons: 5 have reported insufficient knowledge on the topic, 2 no longer held positions in the organizations they represented, 1 was on medical leave and 8 refused to participate. Of these 93 eligible contacts, 90 participants filled in the Delphi form at the first round, which represents 97% response rate. Eight questionnaires were incomplete and were not considered for the analyses, resulting in a total of 82 participants in Round 1. Flowchart for the selection of panelists is presented as Annex 1. In the second round, a total of 68 participants responded which corresponds to a dropout rate of 17%. For a clearer portrayal, please refer to Table 1.

**Table 1:** Delphi panelists' characteristics (%).

	***n***	**%**
**Type of panelist**		
Health professionals or researchers	66	80.5
Stakeholders	16	19.5
**Gender**		
Female	62	75.6
Male	20	24.4
**Age**		
26–45	42	51.2
46–70	40	48.2
**Qualifications**		
Secondary	1	1.2
Bachelor	17	20.7
Master	23	28.0
Doctorate	41	50.0
**Professional activity**		
Research/Teaching	36	43.9
Healthcare	23	28.0
NGOs/Civil society	12	14.6
Governmental Organizations	11	13.4
**Years in the professional activity**		
1–16	44	53.6
17–45	38	46.3
**Municipality of activity**		
Lisbon	63	78.8
Other municipalities	19	21.2
**Field of Science**		
Social sciences	50	61.0
Medical and health sciences	24	29.3
Natural sciences	3	3.7
Humanities	3	3.7
Engineering and technology	1	1.2
**Field of clinical specialty**		
Sexual Health	9	11.0
Reproductive Health	10	12.2
Minority health	5	6.1
Migrant health	4	4.9
Health equity	3	3.7
Social rights	1	1.2
Sexual violence	3	3.7
**Field of Intervention**		
Sexual Health	28	34.1
Reproductive Health	22	26.8
Minority health	23	28.0
Migrant health	16	19.5
Health equity	19	23.2
Social rights	28	34.1
Sexual violence	24	29.3
**Field of Investigation**		
Sexual Health	11	13.4
Reproductive Health	16	19.5
Minority health	10	12.2
Migrant health	17	20.7
Health equity	21	25.6
Social rights	21	25.6
Sexual violence	11	13.4

The following sample characterization concerns the 82 participants who fully completed the first round. The majority of the sample (80.5%) was consisted of experts. More than three quarters (76%) of the panelists were women. Average age was 46 years. Considering their main activity, panelists were classified as health professionals and academics, or stakeholders (associative leaders, members of governmental and non-governmental organizations and political positions). On average, panelists attended their professional positions for 17 years. Academic training was classified according to the Frascati manual (84) and, according to this classification, training in social sciences (61%) and health (29%) predominated as background areas of the participants. The graduates of health sciences were all from the group of researchers; in the graduates of human and social sciences group, a greater diversity was observed, although researchers and health professionals also prevailed. In the graduates of human and social sciences, some diversity was also observed regarding the contexts of activity, with research and teaching predominating. Graduates of health and medical sciences worked majorly in the health sector.

### Indicators

**Figure 4:**
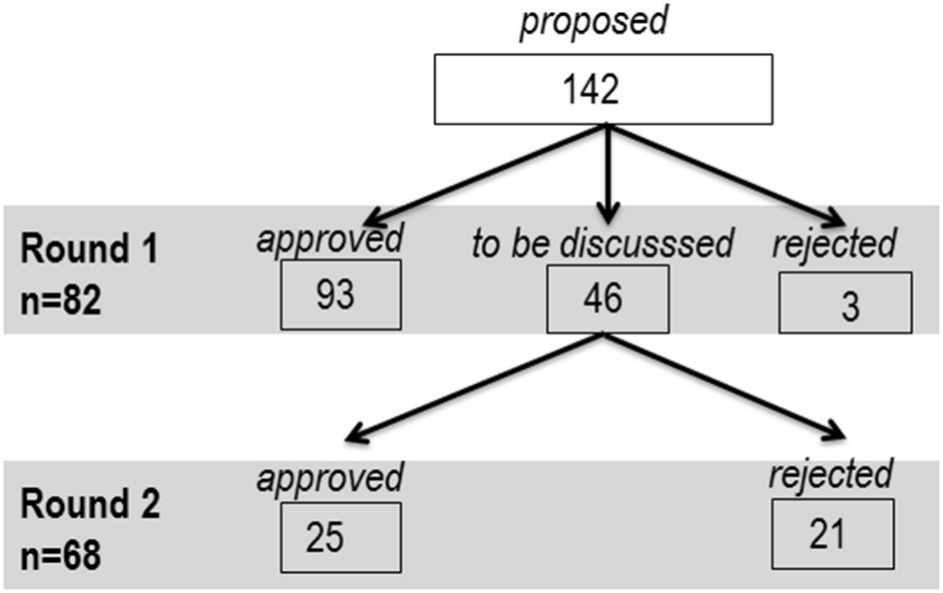
Flowchart for items endorsement/rejection per round and dimension.

From 142 items included in Round 1, 93 items were immediately endorsed to be integrated in the final list, 46 proceeded to Round 2 due to absence of consensus, and 3 were rejected. From the 46 items evaluated in Round 2, 25 were approved and 21 were rejected (Figure 4).

### Item Analysis

From the initial list of 142 proposed items, a consensus was reached on 118 items (83%). The retained items are presented as Annex 2. The distribution of the consensual items by dimension and sub-dimension was the following: *Sexual Health* Indicators (31), *Reproductive Health Indicators* (32), *Social-Structural Factor Indicators* (15), and *Good Practices* (37). A detailed description three groups of indicators and one group of good practices can be found in Table 2.

**Table 2:** Number of indicators proposed, approved or rejected, by dimension and sub-dimension.

	**Proposed (*n*)**	**Approved (*n*)**	**Rejected (*n*)**
*Sexual Health*	*34*	*32*	*2*
Comprehensive education and information	10	10	0
Gender-based violence prevention, support, and care	8	8	0
Prevention and control of HIV and other sexually transmissible infections	11	11	0
Sexual function and psychosexual counseling	5	3	2
*Reproductive Health*	*42*	*33*	*9*
Contraception counseling and provision	8	5	3
Fertility care	9	8	1
Antenatal, intrapartum and postnatal care	21	16	5
Safe abortion care	4	4	0
*Social-Structural Factors*	*27*	*15*	*12*
Cultural and social norms around sexuality	5	3	2
Gender and socioeconomic inequalities	14	6	8
Human rights	5	4	1
Laws, policies, regulations, and strategies	3	2	1
*Good practices*	*39*	*38*	*1*
Total	142	118	24

In Round 1, 93 items reached consensus by the absolute majority (5 > 50% and 1 + 2 < 33.3%) and three items did not reach consensus (1 + 2 = > 20%). In Round 2, the rule of Qualified Majority (5 +4> 75%) was applied, with 25 items being endorsed and 21 items being rejected (Table 3).

**Table 3:** Number of indicators, approved or rejected, by group decision rules and round.

	**Round 1**		**Round 2**	
	**Absolute majority approval**	**Absolute majority rejection**	**Qualified majority approval**	**Qualified majority rejection**
Sexual Health	20	0	12	2
Reproductive Health	28	1	5	8
Social-Structural Factors	12	1	3	11
Good practices	33	1	5	0

An analysis of the mean values calculated by dimension (Table 4) shows that the highest number of endorsed items was obtained in the *Good Practices* dimension and the lowest in the *Social-Structural Factors* dimension. The consensus was higher in the *Sexual Health* and *Good Practices* dimensions and lower in the *Social-Structural Factors*. The percentage of agreement (defined as the percentage of responses in the “*I agree”* or “*I strongly agree”* values of the scale) was higher in the *Good Practices* dimension and lower in *Social-Structural Factors*. Finally, the “no opinion/don't know how to answer” were more frequent in the *Reproductive Health* dimension.

**Table 4:** Mean values for response on Likert scale, Coefficient of variation, Percentage of “agreement” responses and Percentage of “*no opinion”* responses, by dimension.

	**Mean**	**Coefficient of Variation**	**% of Agreement**	**% of No opinion**
Sexual Health	4.31	0.21	84.22	0.86
Reproductive Health	4.23	0.22	80.23	2.86
Social-Structural Factors	3.98	0.25	71.66	1.93
Good Practices	4.47	0.21	88.43	1.38

**Figure 5:**
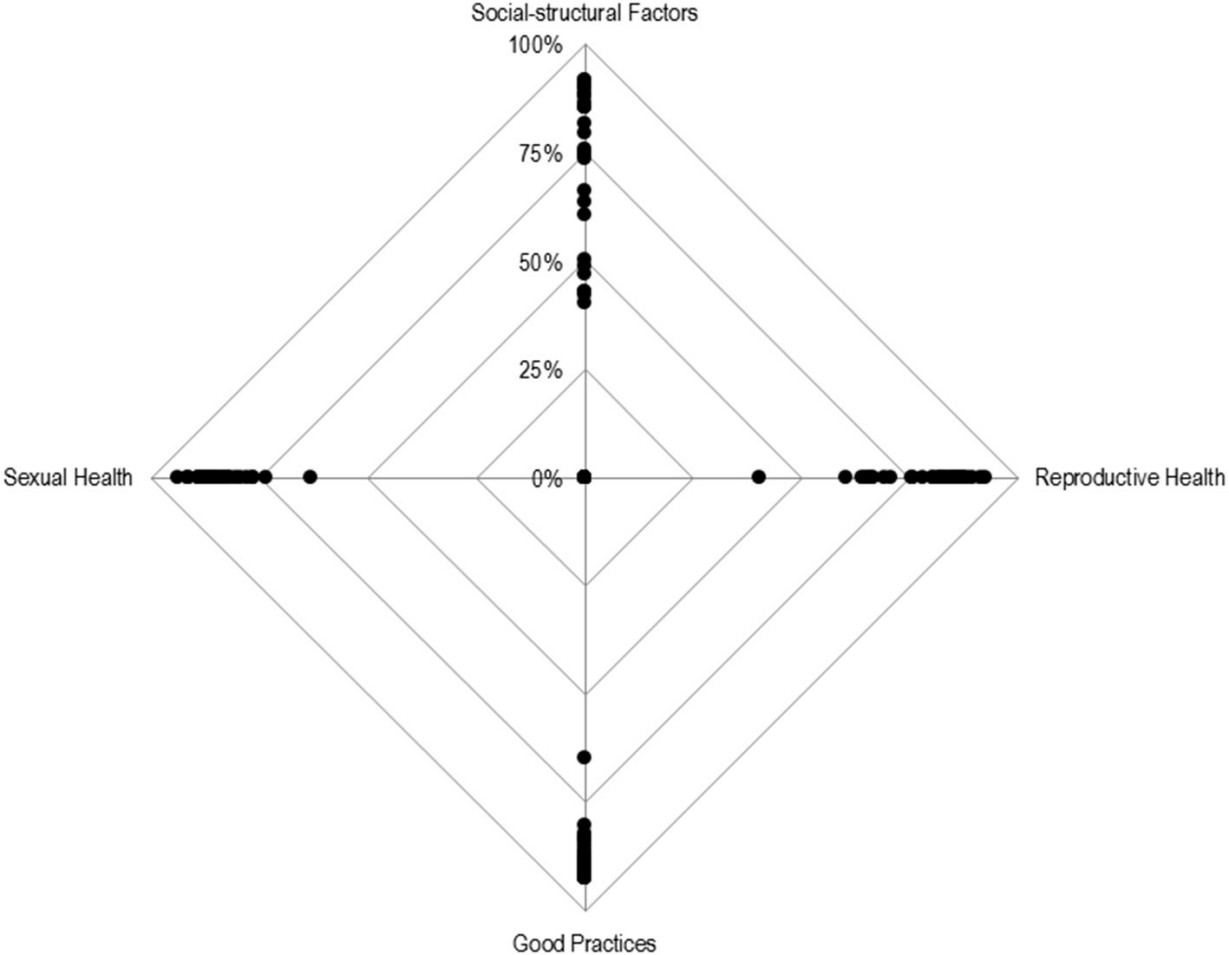
Radar chart for the percentage of responses strongly agree + agree per dimension. Each point represents the percentage of agreement response (“I agree” and “I strongly agree”) for each item, per dimension.

In order to synthesize the collected information, the results for the agreement values at the end of Round 2 are projected in Figure 5. They represent the sum of the two rounds and can be interpreted as an endorsement rate.

Figure 5 shows that there is a high endorsement of items belonging to the *Good Practices* and *Sexual Health* dimensions. There is a high concentration of points near the outer vertex, with only one of the items having an approval rate below 75% (63% precisely). In comparison, the items from the *Reproductive Health* dimension have a lower acceptance rate while the items from the *Social-Structural Factors* demonstrate a greater concentration below 75% of acceptance, with the points scattered along the top vertical line.

In the *Reproductive Health dimension*, the highest endorsement rate was found for items referring to the safe abortion care, such as “*Number of terminations of pregnancy, total and at the option of the woman,” “Number of health services that offer safe termination of pregnancy,”* and “*Number of hospitalizations due to unsafe abortion.”* Regarding *Social-Structural Factors* and *Good Practices*, a regular pattern was not identified in the items with a higher endorsement rate.

### “No Opinion” Rates

**Figure 6:**
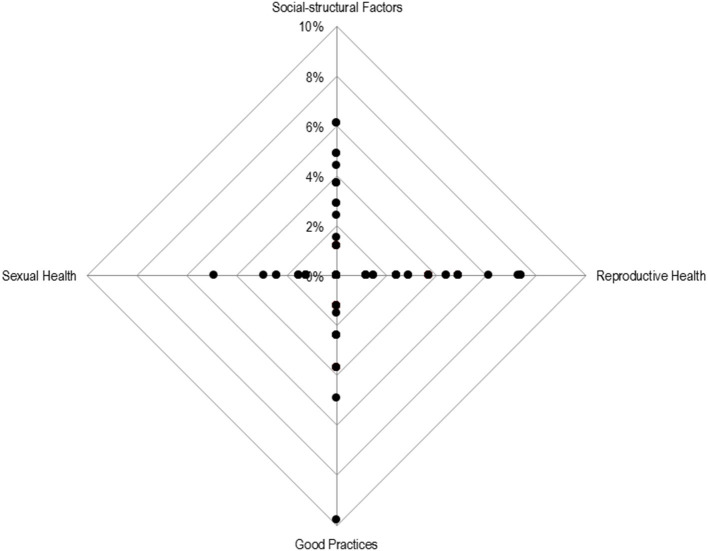
Radar chart for the percentage of no opinions per dimension. Each point represents the percentage of no opinions for each item, per dimension.

“No opinion” rates were used as indicators of unfamiliarity. Considering that there is a pattern of no opinions, they should be considered not missing at random. With these arguments in consideration, Figure 6 represents the distribution of “*no opinion rates*” by dimension. The analysis indicates that “*no opinion*” rates are quite reduced within the *Sexual Health* dimension, while a wider distribution is observed among the *Reproductive Health* dimension and for *Socio-Structural Factors*. Within the *Reproductive Health* dimension, the greatest uncertainties are observed in items related to vaccination, such as “*Number of women of reproductive age who received tetanus vaccine”* and “*Coverage rate of tetanus vaccine by birth cohort.”* Within the *Socio-Structural Factors*, the highest “*no opinion*” rate was observed for the item “*Average age at divorce.”*

### Consensus Analysis

High consensus was observed for all dimensions and in both rounds. The values of the coefficient of variation varied between 0.2 and 0.4 in Round 1, and 0.2 and 0.3 in the Round 2. Although the values are always of high consensus, it can be observed that in Round 1 there is a greater consensus on *Good Practices* and a fewer consensus on *Social-Structural Factors* (Table 5). In the passage to Round 2, the consensus becomes higher in 3 of the 4 dimensions, the exception being *Social-Structural Factors*.

**Table 5:** Mean values for the coefficient of variation by round and dimension.

	**Mean R1**	**Mean R2**
Sexual Health	0.218	0.204
Reproductive Health	0.229	0.225
Social-Structural Factors	0.244	0.276
Good Practices	0.211	0.206

*Coefficient of variation scale ranges from 0 to 1; higher values meaning higher variation*.

**Figure 7:**
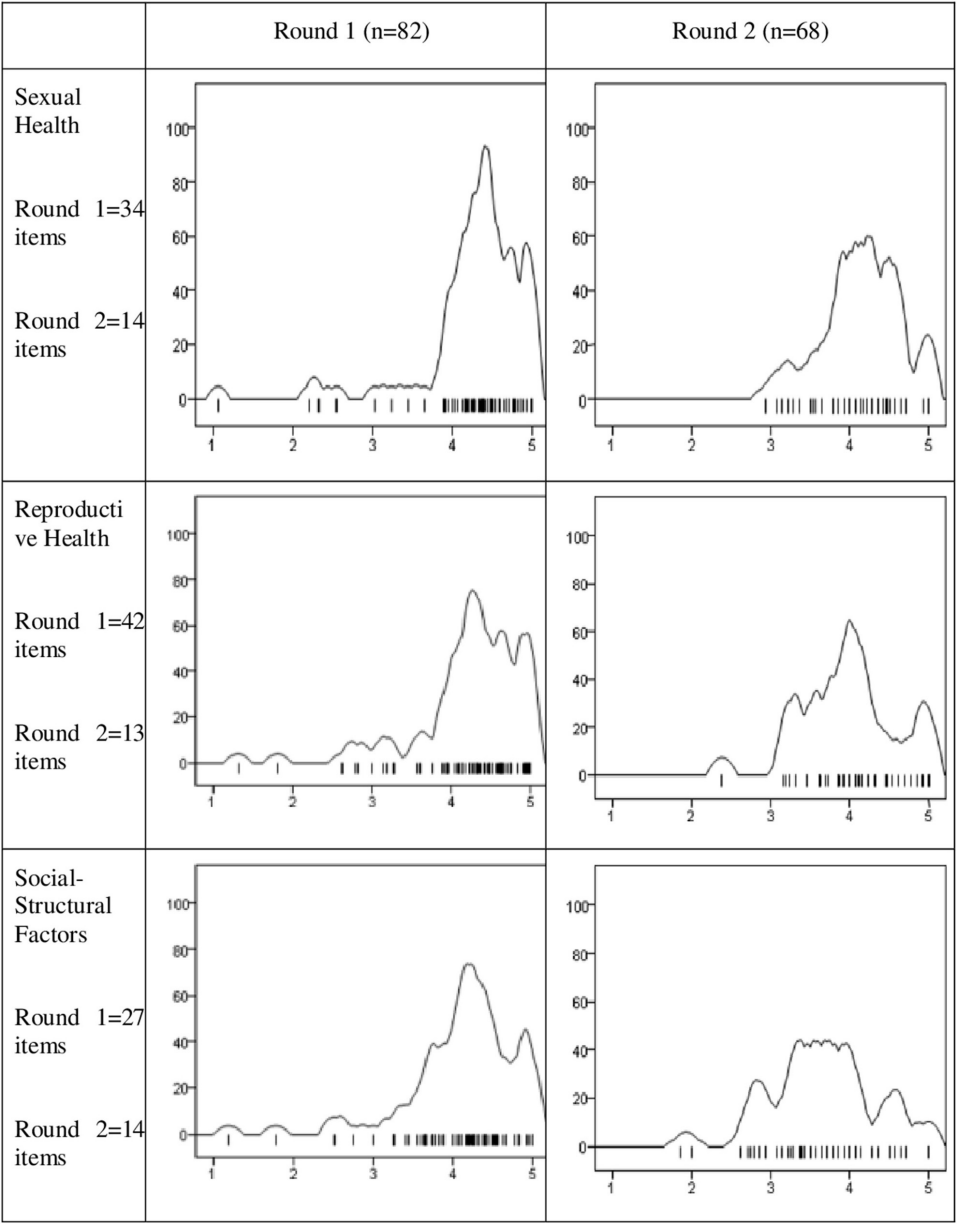
Kernel Density Curves for the panelists mean responses for each round and dimension.

The Kernel Density curves (Figure 7) demonstrate that, in the first round, the distributions are closer to the leptokurtic type (meaning flattened ends and more pronounced mean values), especially regarding the dimensions related to *Sexual Health* and *Good Practices*. The latter is where the greatest concentration on the right is observed, which means greater agreement with the items. In Round 2, the distributions are closer to the Platykurtic type (meaning lower agreement, since there is flatness along the line) and tend to concentrate more on the value 4 than on the 5.

Figure note: Each density curve represents the distribution of the mean of the panelists' responses on a 5-point Likert scale by dimension and round. The curves of the first round relate to the totality of the items that were discussed and include the responses of the 82 participants. The curves corresponding to the second round only refer to the items that were discussed in the second round because they did not have consensus in the first round.

### Changes of Opinion Between Round 1 and Round 2

In order to identify the changes in opinion between the two rounds, a series of McNamar tests was used. After the items' dichotomization (1, 2, or 3 = 0 “*non-agree”*; 4 or 5 = 1 “*agree”*), statistical significance (*p* < 0.05) was observed in six out of the 46 items. Since for each item, the changes can be 2-folded (participants who disagree in R1, agree in R2 and participants who disagree in R1, agree in R2), the most relevant changes were indicated. Significant changes of opinion were observed in two items of the *Reproductive Health* dimension: The “*Number of people who have undergone sterilization,”* 50.0% of the participants who disagreed in R1 changed their position to agreement in R2. Also the “*Number of women who comply with gynecological surveillance recommendations”* in which 72.2% of the participants that disagreed with this item in R1 agreed in R2.The remaining changes of opinion occurred in items within the *Social-Structural Factors* dimension: “*Number of people with health insurance”*—42.5% of the participants who agreed with this item in R1, disagreed in R2; “*Economic well-being”*—35.1% of the participants who agreed with this item in R1, disagreed in R2; “*Gross Divorce Rate”*—42.9% of the participants who agreed with this item in R1, disagreed in R2; and finally the item “*Occupancy rate for Portuguese language courses for foreigners”*—48.8% of the participants who agreed on R1 disagreed on R2.

## Discussion

The aim of this study was to reach consensus on what constitute good practices in the SRH field, with emphasis on SRH equity across native and migrant populations, and to identify the most relevant and inclusive indicators in accordance with the Age Gender and Diversity framework to plan, program, implement, monitor and evaluate SRH in Portugal. Good practices and indicators were grouped into the WHO operational definition of SRH: Sexual Health; Reproductive Health; and Social-Structural Factors (29). The items that received the highest approval rate for each sub-dimension were selected for discussion.

### Sexual Health

Within the Sexual Health dimension which covers areas ranging from comprehensive education and information to sexual function and psychosexual counseling, the items with the highest endorsement rate concern the prevention and control of HIV and other sexually transmitted infections (STI), such as “*Number of new cases (incidence) of sexually transmitted infections,” “Number of HIV/AIDS cases (prevalence),”* or “*Coverage of antiretroviral therapy.”* This occurs even though there were panelists with experience and knowledge in all the other sub-dimensions of sexual health. This is in line with the investment in research intersecting sexual health and migration, where the thematic of the prevention and control of HIV/STI seems to have been receiving more attention, while comprehensive education and information; gender-based violence prevention, support, and care; and sexual function and psychosexual counseling remain understudied areas (85).

Although exhibiting decreasing numbers, HIV/AIDS is a persisting global phenomenon. In Portugal, the cumulative number of people with HIV and AIDS is released annually by the Directorate-General for Health (86) and the PORDATA portal (the Database of Contemporary Portugal official statistics) only discloses data on HIV infection at the AIDS stage. This is one of the cases in which the data is segmented by national origin. In 2018, 61% of new infections were diagnosed in people born in Portugal, 19% in sub-Saharan Africa and 11% in Latin America (87). In addition, the WHO strategy on STIs in people of reproductive age proposes the improvement of the available data by paying special attention to the disaggregation by sex and age groups (88).

Previous studies have also highlighted the need for on-going monitoring of risk behaviors, STIs, and accessing services among migrant populations, as well as further research to help understand its intersecting inequities (89). It is important to identify key populations (including migrants) to be targeted with tailored HIV prevention activities and treatment options, as well as services that provide care and support based on the recipients' different backgrounds and needs (90, 91). Regarding the *Comprehensive education and information* sub-dimension, the panel of experts endorsed the importance of the “*Number of people with levels of sexual health literacy considered adequate.”* An adequate level of health literacy would contribute to making informed decisions, which contribute to an increase in migrants' health and empowerment (92, 93).

Within the sub-dimension *Gender-based violence prevention, support and care*, high endorsement was found for the item “*Number of people who correctly identify gender and sexual violence.”* Monitoring gender violence and reducing its structural risk factors remains a vital public health priority (94). Although indicators on gender violence and sexual violence in Portugal have been published annually since the beginning of the millennium (e.g., APAV—Portuguese Association for Victim Support—which has a specialized support unit for migrant and discrimination victims) (95), there is lack of information regarding the extent to which the population is aware of the forms gender and sexual violence can take. Sexual violence victimization has been associated with a broad range of health and risk behaviors, including posttraumatic stress disorder, depression, eating disorders, substance use, smoking, and poor self-rated health (28). In Portugal, despite the presence of organizations such as the National Observatory of Violence and Gender that conduct victimization surveys, this dimension (the correct identification of gender violence) is not properly accounted for. It should be noted that both gender and sexual violence must be carefully defined, to avoid generalizations or create an overgeneralized concept not allowing the identification of different potential forms of violence. The need for sexual and gender-based violence conceptualization is well-illustrated in a recent study in the context of European asylum reception centers, which showed a disparity between what is, or what is not considered a violent behavior among residents and professionals, the latter considering more acts as violence then the former (96). Public health policies should be adapted to the cultural and structural context, and for that comparing sexual and gender-based violence conceptualization between migrants and hosting population is crucial. The development, implementation and monitoring prevention programs in this area would benefit from a comprehensive societal conceptualization of sexual and gender-based violence considering the influences of individual, relational, community, and societal factors (96).

In addition, within the sub-dimension *Gender-based violence prevention, support and care*, the item “*Number of reports of obstetric violence”* deserves attention. Although sexual and reproductive rights are protected under Portuguese law, obstetric violence is an existing phenomenon that currently lacks a legal framework and remains difficult to quantify. The survey “Childbirth Experiences in Portugal,” carried out by the Portuguese Association for Women's Rights in Pregnancy and Childbirth, collected responses of more than 3,800 women and revealed that 43.5% of the women surveyed did not have the desired delivery, however very few of them filed complaints. The proper identification of obstetric violence would contribute with a 2-fold benefit to SRH: (1) it is a form of gender-based violence that would be identified and, potentially prevented; and (2) based on women's experiences and perceptions during childbirth, as well as on the normative pattern of obstetric management it would allow the provision of a physically and mentally healthy birth (97, 98).

Inequities in the quality of care must be understood in light to the intersecting challenges migrant women face due to language difficulties, lack of familiarity with healthcare systems, and discriminatory attitudes (99).

Within the sub-dimension “*Sexual function and psychosexual counseling,”* the items with the highest endorsement were “*Number of new cases (incidence) diagnosed with sexual dysfunction,”* and “*Number of people who consider that have a healthy sexuality.”* Sexual dysfunctions are a multifaceted phenomenon that can be understood as the reason that prevents individuals from experiencing satisfaction from sexual activity (29). Although there are several scales already validated among Portuguese samples (88, 100), longitudinal studies that can assess the evolution of prevalence of various sexual dysfunctions are still missing. Additionally, more knowledge is needed concerning the individual sexual well-being of the Portuguese population, using positive indicators of sexual health such as sexual satisfaction (101, 102). An assessment of subjective sexual well-being, defined as the cognitive and emotional assessment that each person makes of their sexuality (103), was applied as part of an international study—the Global Study of Sexual Attitudes and Beliefs (104) but Portugal did not participate. It would be important to replicate the study in the Portuguese population, including migrants and contribute to overcome the scarcity of data intersecting sexuality and migration.

### Reproductive Health

Reproductive Health dimension embraces the WHO definition of reproductive health and rights, such as the right make a free and responsible decision on the number, spacing, and timing of their children; ability to obtain the appropriate information and means to make such a decision; and the right to decide on reproduction without threat of discrimination, coercion, and violence (105). Despite the significant reduction in the number of cases of unwanted pregnancy in the last two decades worldwide, the phenomenon continues as a significant burden globally, with ~16 million (11%) of all births worldwide attributed to young women aged 15–19 years (106). In Portugal, the interruption of pregnancy on women's request can be seen as a way of regulating fertility in order to limit births of unwanted pregnancies (107). A study revealed that more than 95% of all interruptions of pregnancy performed in the country, were performed in hospital settings (86, 108). In 2018, 20% of all women who interrupted their pregnancy in Portugal were foreigners (i.e., 3.098 in 11.827) (86, 108, 109). Although there are several causes of unintended pregnancy, one of the most important tools that can help in preventing them is the timely use of emergency contraception and access to primary health facilities that provide family planning services. According to the WHO, all women and girls at risk of an unintended pregnancy have the right to access emergency contraception and these methods should be routinely included within all national family planning programs (109, 110). As an indicator, the number of sold emergency contraception pills can inform on the number of terminated unwanted pregnancies, but also may point to the ineffectiveness of regular contraception. Despite its relevance, currently there is no official data available in Portugal.

The items “*Number of women who comply with gynecological surveillance recommendations”* and “*Number of family planning users who were counseled, referred or treated for infertility”* had the highest level of endorsement by the expert panel within the “*Fertility care”* sub-dimension.

Ensuring universal access to SRH services is incorporated in Target 3.7 of the United Nations' Sustainable Development Goals (SDG). Refugees, migrant women and children are at particular risk of being excluded in achieving this target, since they hold a higher chance of maternal death and maternal near-miss events (111, 112). In this context, gynecological surveillance is important to prevent potential complications, with the recommended number of annual visits depending on the woman's age and the existence of previous problems. In Portugal, the relevant data can only be obtained at the aggregate level (and excludes the entire private sector). Therefore, the alternative would be to resort to population surveys. The second item with highest approval rate in this sub-dimension, concerning the identification and management of infertility, must be analyzed in conjunction with others, such as the quality of services received. Currently, public and private offers for infertility treatments are available. However, several negative beliefs and representations block access to these services. A survey on this topic of a representative sample of the Portuguese population (113) estimated that 9.8% of women aged between 25 and 69 years had already had problems with pregnancy, of which 43.4% had consultations for reasons of infertility. The number of people doing infertility treatments can serve as an orientation point of the number of people who, regardless of constraints, are referred in order to enjoy a desired pregnancy.

Within the “*Antenatal, intrapartum and postnatal care”* sub-dimension, the expert panel considered as most important to focus on “*Gestational age of women at the first consultation of Gynecology-Obstetrics”* and “*Maternal mortality rate, by cause.”* The reduction in the global maternal mortality rate is part of the sustainable development goals for 2030 (24, 114). According to the WHO although the maternal mortality rate and rate of complications in childbirth in Portugal has been reduced, the global maternal mortality rate is still unacceptably high. The high number of maternal deaths in some areas of the world reflects inequity in the access to quality health services and highlights the gap between rich and poor countries. A distinction is also made between “maternal mortality” (death of women during pregnancy or within 42 days after termination of pregnancy, excluding external causes) and “late maternal mortality” (when it concerns obstetric causes, direct or indirect, after 42 days, and less than a year after termination of pregnancy). It is proposed (second most consensual item) that this indicator should be disaggregated by the main cause of death in order to better understand this multifaceted phenomenon and the areas of intervention. The recommendations of the Portuguese General Directorate of Health imply that a normal pregnancy should have at least six consultations that can identify potential risk factors and needs for intervention. However, the proportion of pregnant women who act in line with this recommendation is still unknown.

Additionally, within the “*Safe abortion and care”* sub-dimension, the importance of “*Number of terminations of pregnancy, total and at the request of the woman”* was highlighted. The situation of induced abortion has changed markedly over the past few decades, with abortion being legalized and its rates dropping in many developing countries in the world (115). The Guttmacher Institute report shows that abortion rates are similar in countries where abortion is highly restricted and where it is broadly legal (116). In Portugal, the Directorate-General for Health compiles the number of pregnancy interruptions that occur in public and private health facilities. The reports with these numbers contemplate time series and present a characterization of the women who utilized this service (117). As in other cases, it is an ambivalent indicator, especially when it comes to interruptions that occur at the request of women.

Evidence gathered by the international research collaboration ROAM (reproductive outcomes and migration) from 20 countries including Portugal shows that culturally diverse guidelines are needed to individualize antenatal care and promote optimal maternal-fetal health outcomes across cultural groups (118, 119). Further research is needed to identify and understand specific vulnerabilities and subsequent action is needed to address the intersecting inequities.

### Social-Structural Factors

*Social-Structural Factors* dimension covers items ranging from *Cultural and social norms* a*round sexuality* to *Laws Policies, regulations, and strategies*.

The two highest endorsed items in the “*Cultural and social norms around sexuality”* sub-dimension were “*Number of complaints of female genital mutilation”* and “*Number of people who report that their partner's sexual pleasure is important for the quality of the relationship.”*

In Portugal, Female Genital Mutilation (FGM) is considered an autonomous crime according to article 144 A of the Criminal Code of 2005. The applicable penalty is 2 to 10 years in prison. The currently existing numbers of FGM (64 cases in 2018 and 129 cases in 2019) are the result of cases identified by health professionals, who received training under the “*Healthy Practices”* project, which covered groups of health centers with the highest number of women at risk. Although no reliable data exist, estimates point to 6,576 women living in Portugal already subjected to FGM (120).

Regarding the second item, no reliable data exist, as sexual pleasure is under-researched and there is only one available measure addressing this sexual health dimension (115, 116). Furthermore, the existing research does not take a partner-centered approach (121). Sexual pleasure is at the heart of sexual rights advocacy (122, 123) and our results support this view. The inclusion of items related to interpersonal pleasure would allow a more complex and accurate picture on the interpersonal nature of sexual pleasure.

Within the *Gender and socioeconomic inequalities* sub-dimension, the highest endorsement was found in the items “*Rate of adherence to cervical cancer screening”* and “*Paternity leave utilization rate.”* Strategies to reduce inequalities in adherence to cervical cancer screening are needed, to allow timely diagnosis and improve the sexual life of all women diagnosed after treatment. These include cultural competence in healthcare and having cervical cancer screening information linguistically and culturally adapted (124, 125). Within the scope of the National Program for Oncological Diseases of the Portuguese General Directorate of Health, data on the rate of adherence to cervical cancer screening were released. The applied measure is “*Total Number of Women Tracked/Number of Women Invited.”*

The second most consensual item provides an interesting insight into parenting. It is important to know the proportion of fathers who want or have the possibility to take full paternity leave, also because the stay of both parents during the initial period promotes a healthy child development and less overload of domestic tasks for the recent mothers, thus improving their postpartum condition (126, 127). According to OECD data (128), Portugal is one of the countries in this group with the longest duration of paternity leave (21 weeks in 2015), and the share of men among parental leave users in Portugal, as well in some Nordic countries, goes up to 40% or more. Fathers-only Parental leave (formerly Paternity leave) is a relatively recent right, since it was non-existent until the year 1999. Using data from the Social Security data and the number of births available on the PORDATA portal, the Observatory of Families and Family Policies (129), found an increasing tendency with 68% of fathers using their right to paternity leave in 2019.

Research has shown that besides attitudes toward gender roles within the family sphere, the level of knowledge about the parental leave system, the vulnerability on the labor market, and non-universal eligibility are major factors explaining migrant-native differentials in parental leave use. In this sense, parental leave policies need to avoid perpetuating labor market disadvantages by limiting support for work–family reconciliation (130, 131). Further research is needed on the differences in parental leave use between different groups of parents. In the *Human rights* sub-dimension, the expert panel considered important to address the “*Number of complaints for discrimination based on gender identity and sexual orientation.”* Since 2013, the ILGA Portugal Association's Observatory of Discrimination Based on Sexual Orientation and Gender Identity—Lesbian, Gay, Bisexual, Trans, and Intersex Intervention collects, analyzes, and disseminates data on complaints of discrimination (132). However, the collected data lacks disaggregation to provide adequate assessment of this issue (133).

Studies have shown that transgender migrants and migrants who engage in sex work also face higher risk for HIV infection (89). The UNAIDS Gap Report highlights how migrants who engage in sex work face a double stigma because of their immigration status and their engagement in sex work. Adding the fact that stigma and discrimination of living with HIV amplifies their risk of experiencing violence and the barriers to accessing services (134). Of most importance for practice, is the fact that the characteristics of the country of origin and destination (such as access to healthcare, social protection, and social exclusion) influences migrants' risk of HIV infection (134). In the final sub-dimension in this category, “*Laws, policies, regulations and strategies*,” the expert panel considered “*Number of Local Support Centers for the Integration of Migrants (CLAIMs) available to the migrant population”* and “*Percentage of government spending on health, directed at SRH”* to be of highest importance.

According to a recent study, Portugal is one of the three European Union countries (together with Ireland and Spain) that propose their largest range of policies aiming at improving access to healthcare services for migrants (135). In this context, Portugal has founded CLAIMs and has also made efforts toward securing a specific budget for these relevant issues. CLAIMs were founded in 2003 and they help in “*regularization, nationality, family reunification, housing, voluntary return, work, health, education, among other issues of daily life”* (136). The Portuguese CLAIMs network includes already more than 100 centers, provides information and assistance. In the context of the second most consensual item of this sub-dimension, the percentage of government expenditure that is directed to health is available from the Portuguese Directorate-General for Budget and from the Ministry of Finance, with a proposal for a separate breakdown for the Division of Sexual, Reproductive, Child and Youth Health (DSSRIJ).

A final issue deserves attention: the answer “*no opinion”* can be interpreted as an indicator of the areas in which further intervention in terms of dissemination and training may be needed (137).

### Good Practices

Finally, the most highly endorsed *Good Practices* by the expert panel were: (1) “*Existence of procedures in healthcare units that guarantee the informed choice in SRH”*; (2) “*Health facilities, goods, information and health services related to SRH must be accessible to all individuals and groups without discrimination and free from obstacles”*; (3) “*Existence of evidence-based SRH counseling services”* and (4) “*Existence of laws and regulations that guarantee full and equal access to SRH care.”*

In the *Good Practices* dimension, apart from the low “*no opinion”* rate, there is an observed outlier concerning the “*Greater coverage of the reasons why abortion is permitted”* item. In Portugal, two referendums were needed to stablish that voluntary abortion was no longer illegal when performed up to the 10th gestational week in official or officially accredited health services since 2007, thus voluntary abortion remains a fracturing issue in Portuguese society (108, 138).

### Strengths and Limitations

This study represents a contribution toward the identification of country-based relevant indicators on the SRH and rights to improve health and well-being for all (139). This research has some limitations to consider. First, although the Delphi panel was consisted of a variety of experts and stakeholders, the convenience (snowball) sampling method may influence the transferability of experts' opinions to that of the wider community of professionals that work in the relevant field and with the population of interest to this study. Even so, an effort was made in order to be the most comprehensive as possible in sampling in order to have a varied sample of panelists. Another limitation of this study is the reduced participation of stakeholders representing migrant communities. Although invitations and reminders were made to various actors in this area, this was clearly the area of intervention in which less adherence was felt. Further studies are needed to investigate and understand the preferences of migrants and their families on how relevant SRH issues should be promoted. Still another limitation linked with the sample of panelists, regards the fact that it included more specialists than stakeholders. On the other hand, a main strength of this study remains on the inclusion of a variety of experts and stakeholders, with diverse professional backgrounds and with extensive experience, underlining their potential for a strong contribution in the area.

Despite the fact that the date of the implementation of the second round (12 to 31 March, 2020) coincided with the first confinement due to COVID-19 pandemic, within the scope of the first state of emergency, declared on 19 March 20 by the council of Portuguese ministers, response rate exceeded the recommend 70% rate as necessary to maintain rigor (41).

Although the findings of this study are intended to be formative rather than definitive, the final set of items is valid and consistent with a range of important dimensions related to SRH areas, and also diverse and inclusive to enable monitoring inequalities.

### Future Recommendations for Research and Action

Results highlight the importance of identifying and understanding the origin of health inequalities, inequities, and monitoring the impact on SRH and rights between ethnic/racial minorities and migrant groups. Addressing the social determinants of health inequalities and inequities holds the potential to raise awareness to design appropriate interventions both in terms of access to healthcare and quality of SRH services.

Findings can serve for inspiration to the multiple actors in the field of SRH who wish to protect and promote SRH human rights by building operational links between principles and realities.

In 2007, the ROAM international research collaboration and EURO-PERISTAT project developed an international Delphi survey to recommend migration indicators for national and international monitoring. A strong consensus was attained to include firstly country of birth and secondly length of time in the country in core perinatal health indicator sets. Specific studies were also recommended to complement routine data collection on three other indicators of migration: migration status, receiving-country language capacity, and maternal parents' place of birth as proxy for ethnicity (45). These recommendations remain up to date and should be expanded to the overall SRH issues and across life course and populations to effectively reduce SRH inequities between migrant and receiving-country populations.

The Academic Network for Sexual and Reproductive Health and Rights Policy (ANSER) is a global platform for SRH and rights policy research, education and healthcare delivery that addresses the gap between research and policy in this area. It is a good example of how SRH research findings can be translated into feasible policy and practice by engaging effectively stakeholders at different stages of the research cycle and by taking into account existing and changing political contexts and priorities (140).

Findings can serve as a starting point to awareness-raising actions on the cultural, socioeconomic, geopolitical and legal environment diversity that forms the context for people's lives in different settings and which influences SRH outcomes. They can also serve the basis for providing training to health professionals toward an improved focus on migrants' needs, and effective communication practices (141).

## Conclusion

This study reinforces the need to address the wide variation of national contexts regarding policy measures to protect migrants' SRH and rights and ensuring their access to basic and essential services—with special emphasis on sexual education, as well as sexual and reproductive justice. The Delphi method, as performed in this study, provided avenues that can be used by the healthcare system to engage in better informed decisions and, more importantly, inclusive and integrative strategies regarding SRH equity. Given the global COVID-19 pandemic, the findings are of special importance since the existing achievements to promote equal access to healthcare and decrease the risk of healthcare-related inequities, were undermined. Results can enable the health systems to adapt to the needs of the migrant population and thus ensure effective and efficient deployment of SRH care structures and processes within the context of inclusive and integrated care. As envisaged throughout the paper, this can be achieved by using the life course approach to plan, program, implement, monitor, and evaluate the relevance of SRH indicators of the populations and across life course.

## Data Availability Statement

The raw data supporting the conclusions of this article will be made available by the authors, under request.

## Ethics Statement

The study was approved by the Ethics Committee of the Centro Académico de Medicina de Lisboa (CAML). All participants received an online consent form, together with the questionnaire forms, informing about the project aims and their rights (e.g., procedures, voluntary non-gratified participation, data confidentiality, dropout option with no consequences.

## Author Contributions

VA, OS, and AV designed the study and wrote the protocol with inputs from PC, SP, PP, AC, and FM. PC, VA, OS, and AV did the initial survey form, with the review of PC, SP, PP, AC, and FM. VA and PC were responsible for the recruitment of participants and data collection. PC performed data analysis and interpreted the results with the review of VA, OS, and AV. PC, VA, and MS-P wrote the first draft of the manuscript. All authors reviewed, contributed to the article and approved the submitted version.

## Conflict of Interest

The authors declare that the research was conducted in the absence of any commercial or financial relationships that could be construed as a potential conflict of interest.
