# Supplementary material for: Reducing Sexual and Reproductive Health Inequities Between Natives and Migrants: A Delphi Consensus for Sustainable Cross-Cultural Healthcare Pathways
Source: Front Public Health. 2021 May 13;9:656454. doi: 10.3389/fpubh.2021.656454 (PMC8155376; doi:10.3389/fpubh.2021.656454)
Supplement: Supplementary file 1 [file Data_Sheet_1.docx]

Annex 1. Flowchart for the selection of panelists


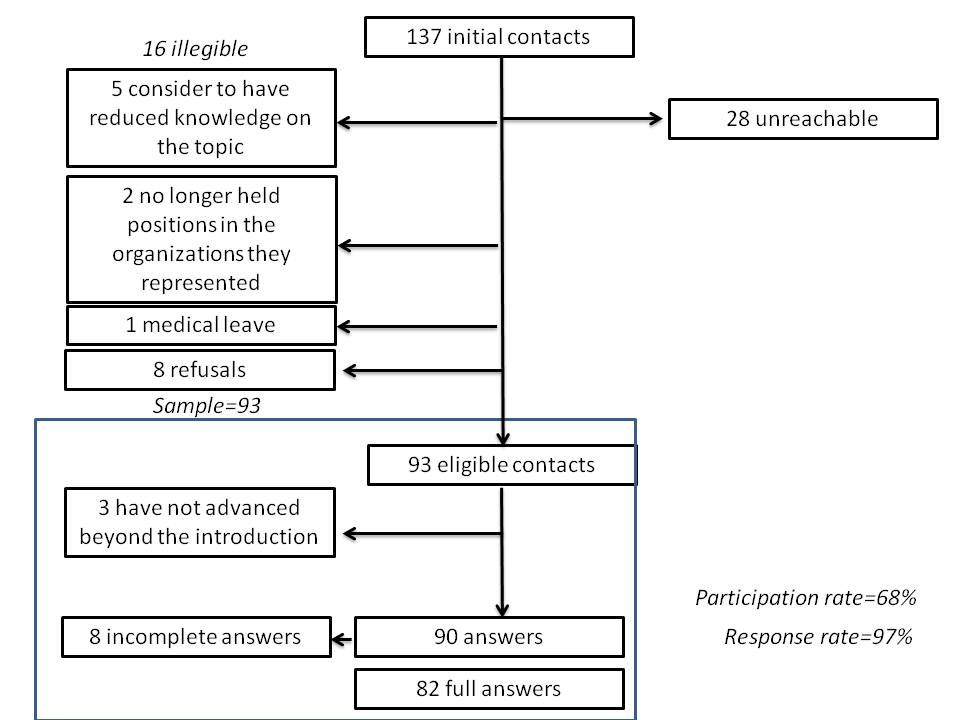


**Annex 2. Consensual items regarding Sexual Health, Reproductive Health, Social-Structural Factors and Good Practices**

|  | **Mean** | **Coefficient of Variation** | **% of agreement** | **no opinion %** |
| --- | --- | --- | --- | --- |
| **SEXUAL HEALTH** |  |  |  |  |
| *Comprehensive education and information* |  |  |  |  |
| **Number of people with levels of sexual health literacy considered adequate** | **4.31** | **0.19** | **86.76** | **0.00** |
| Number of students who received sex education within the education system, by cycle of studies | 4.21 | 0.22 | 83.82 | 0.00 |
| **Number of adolescents that got an appointment for family planning/Sexual and Reproductive Health consultation in primary health care units** | **4.31** | **0.21** | **86.42** | **1.20** |
| Number of health professionals trained in the provision of Sexual Health education and information | 4.32 | 0.19 | 85.29 | 0.00 |
| Number of health professionals trained in the provision of clinical services related to Sexual Health | 4.32 | 0.21 | 82.35 | 0.00 |
| Number of joint initiatives between schools and other entities on Sex Education themes | 4.04 | 0.21 | 76.47 | 0.00 |
| Number of school establishments with health professionals who provide Sexual Health counseling | 4.25 | 0.18 | 82.09 | 1.50 |
| Number of health professionals’ consultation on themes related to Sexual Health | 4.27 | 0.17 | 83.58 | 1.50 |
| Number of health units with health professionals trained in Sexual Health | 4.24 | 0.24 | 80.49 | 0.00 |
| Expenditures of health services with training of health professionals in Sexual and Reproductive Health | 3.93 | 0.23 | 76.47 | 0.00 |
| *Gender-based violence prevention, support and care* |  |  |  |  |
| **Number of people who correctly identify gender and sexual violence** | **4.35** | **0.24** | **82.72** | **1.20** |
| Number of people who report knowing what to do in order to report gender and sexual violence | 4.40 | 0.21 | 85.37 | 0.00 |
| Number of health professionals trained to detect signs of gender and sexual violence | 4.46 | 0.23 | 87.80 | 0.00 |
| Number of police forces trained to respond appropriately in situations of gender and sexual violence | 4.42 | 0.23 | 85.19 | 1.20 |
| Number of health facilities providing medical, psychological or other services needed by victims of gender and sexual violence | 4.47 | 0.20 | 88.89 | 1.20 |
| **-** | **4.33** | **0.20** | **85.00** | **2.40** |
| Number of referrals and complaints to victim support services, for reasons of gender and sexual violence | 4.43 | 0.20 | 86.59 | 0.00 |
| Number of complaints of gender and sexual violence | 4.36 | 0.20 | 86.42 | 1.20 |
| *Prevention and control of HIV and other sexually transmissible infections* |  |  |  |  |
| Number of people who correctly identify ways to prevent sexual transmission of HIV | 4.52 | 0.19 | 89.02 | 0.00 |
| Number of people who during the last year have been involved in risky practices (buying and selling sex, unprotected sex, injected drug use) | 4.28 | 0.21 | 82.09 | 1.50 |
| Number of HIV-positive people who were offered treatment | 4.44 | 0.19 | 88.89 | 1.20 |
| Number of HIV-positive people advised on Sexual and Reproductive Health rights, including family planning | 4.37 | 0.22 | 87.65 | 1.20 |
| Number of people subject to pre-exposure prophylaxis | 4.36 | 0.22 | 88.46 | 4.90 |
| Coverage of antiretroviral therapy | 4.51 | 0.17 | 91.25 | 2.40 |
| HPV vaccine coverage rate | 4.35 | 0.22 | 85.37 | 0.00 |
| **Number of HIV/AIDS cases (prevalence)** | **4.54** | **0.16** | **91.46** | **0.00** |
| Number of cases of mother-to-child transmission of HIV | 4.37 | 0.22 | 83.95 | 1.20 |
| **Number of new cases (incidence) of sexually transmitted infections** | **4.60** | **0.17** | **93.83** | **1.20** |
| Number of family planning units offering HIV counseling and testing | 4.41 | 0.22 | 87.65 | 1.20 |
| *Sexual function and psychosexual counselling* |  |  |  |  |
| **Number of people who consider that have a healthy sexuality** | **4.06** | **0.21** | **79.41** | **0.00** |
| Number of people who want to discuss sexuality issues with health care providers | 4.04 | 0.24 | 73.53 | 0.00 |
| Number of people who report high sexual satisfaction | 3.79 | 0.24 | 63.24 | 0.00 |
| Number of health care providers who proactively advise their patients about their health or sexual well-being | 4.10 | 0.19 | 77.94 | 0.00 |
| **Number of new cases (incidence) diagnosed with sexual dysfunction** | **4.27** | **0.17** | **87.88** | **2.90** |
| **REPRODUCTIVE HEALTH** |  |  |  |  |
| *Contraception counseling and provision* |  |  |  |  |
| Number of sexually active people who consistently use contraception | 4.39 | 0.18 | 85.37 | 0.00 |
| Number of people who have undergone sterilization | 3.84 | 0.23 | 65.67 | 1.50 |
| Percentage of people of childbearing age using modern contraceptive methods (male and female sterilization, pill, IUD, injectables/implants, condoms, foam/diaphragm/gel), by type | 4.32 | 0.20 | 85.19 | 1.20 |
| Number of women using mobile phone applications to monitor their menstrual cycle | 3.41 | 0.33 | 40.51 | 3.70 |
| **Number of morning-after pills sold or made available** | **4.40** | **0.18** | **87.50** | **2.40** |
| Number of condoms distributed throughout the country | 3.90 | 0.25 | 70.59 | 0.00 |
| **Number of primary health facilities that provide family planning services** | **4.43** | **0.22** | **86.59** | **0.00** |
| Number of health facilities that offer information on different contraceptive methods | 4.40 | 0.21 | 85.37 | 0.00 |
| *Fertility care* |  |  |  |  |
| **Number of family planning users who were counseled, referred or treated for infertility** | **4.38** | **0.20** | **86.25** | **2.40** |
| Number of people who have used infertility treatments | 4.41 | 0.21 | 85.19 | 1.20 |
| **Number of women who comply with gynecological surveillance recommendations** | **4.38** | **0.15** | **89.71** | **0.00** |
| Number of live births resulting from infertility treatments | 4.26 | 0.24 | 78.21 | 4.90 |
| Number of in vitro fertilizations (IVF) per year | 4.27 | 0.22 | 82.28 | 3.70 |
| Synthetic Fertility Index | 4.14 | 0.27 | 75.64 | 4.90 |
| Number of new cases diagnosed with infertility | 4.20 | 0.19 | 80.30 | 2.90 |
| Ideal age for the birth of the first child | 3.75 | 0.24 | 64.18 | 1.50 |
| Maternal mortality rate, by reason | 4.38 | 0.26 | 85.00 | 2.40 |
| *Antenatal, intrapartum and postnatal care* |  |  |  |  |
| Number of people exposed to information on maternal and neonatal care | 4.13 | 0.17 | 82.35 | 0.00 |
| **Gestational age of women at the first consultation of Gynecology-Obstetrics** | **4.38** | **0.18** | **88.61** | **3.70** |
| Number of women of reproductive age who received tetanus vaccine | 3.79 | 0.26 | 60.32 | 7.40 |
| Number of women with high-risk pregnancies with hospital deliveries | 4.28 | 0.24 | 82.50 | 2.40 |
| Time interval between pregnancies for women who have been through more than one pregnancy | 3.81 | 0.23 | 64.71 | 0.00 |
| Coverage rate of whooping cough vaccine in pregnant women | 3.88 | 0.27 | 66.15 | 4.40 |
| Coverage rate of measles and rubella vaccine in pregnant women | 4.06 | 0.23 | 75.76 | 2.90 |
| Coverage rate of tetanus vaccine in pregnant women | 3.91 | 0.25 | 69.23 | 4.40 |
| Number of episodes of violence during pregnancy reported to police forces and/or health professionals | 4.32 | 0.23 | 87.34 | 3.70 |
| Number of premature births | 4.44 | 0.20 | 87.18 | 4.90 |
| Number of live births with low weight | 4.42 | 0.20 | 86.84 | 7.30 |
| Birth rate | 4.37 | 0.25 | 82.28 | 3.70 |
| Number of live births per year | 4.31 | 0.26 | 80.77 | 4.90 |
| Births: total and in health facilities | 4.47 | 0.20 | 87.34 | 3.70 |
| Mother's mean age at birth of first child | 4.27 | 0.23 | 83.33 | 4.90 |
| Average age of mothers at birth of children | 4.15 | 0.20 | 86.76 | 0.00 |
| Number of cases of complications during childbirth | 4.37 | 0.24 | 83.54 | 3.70 |
| Coverage rate of tetanus vaccine by birth cohort | 3.94 | 0.26 | 66.67 | 7.40 |
| **Maternal mortality rate, by reason** | **4.59** | **0.20** | **87.50** | **2.40** |
| Number of women using postpartum contraception methods | 4.28 | 0.22 | 82.05 | 4.90 |
| Number of mothers who adhered to postpartum obstetrics and gynecology consultations | 4.27 | 0.23 | 84.42 | 6.10 |
| *Safe abortion care* |  |  |  |  |
| Number of health services that offer safe termination of pregnancy | 4.57 | 0.17 | 91.36 | 1.20 |
| **Number of terminations of pregnancy, total and at the option of the woman** | **4.60** | **0.17** | **92.59** | **1.20** |
| Number of women who are advised to have a family planning method after termination of pregnancy | 4.35 | 0.22 | 85.19 | 1.20 |
| Number of hospitalizations due to unsafe abortion | 4.54 | 0.19 | 91.36 | 1.20 |
| **SOCIAL-STRUCTURAL FACTORS** |  |  |  |  |
| *Cultural and social norms around sexuality* |  |  |  |  |
| Number of people who consider their sexual well-being fundamental to their global well-being | 4.01 | 0.20 | 73.53 | 0.00 |
| **Number of people who report that their partner's sexual pleasure is important for the quality of the relationship** | **4.09** | **0.17** | **79.41** | **0.00** |
| Number of sexual partners in the last year | 3.74 | 0.31 | 63.24 | 0.00 |
| **Number of complaints of female genital mutilation** | **4.53** | **0.20** | **89.87** | **3.70** |
| Average age at first sexual intercourse | 3.96 | 0.24 | 75.00 | 0.00 |
| *Gender and socioeconomic inequalities* |  |  |  |  |
| Number of people with health insurance | 3.15 | 0.34 | 40.00 | 4.40 |
| Evaluation of users' satisfaction with the NHS health services, by sectors (primary, secondary and tertiary) | 4.32 | 0.21 | 81.48 | 1.20 |
| Economic well-being | 3.65 | 0.29 | 60.29 | 0.00 |
| Unemployment rate | 4.34 | 0.21 | 86.25 | 2.40 |
| Marriages between people of the opposite sex and people of the same sex | 3.49 | 0.31 | 50.00 | 0.00 |
| Average age at the time of marriage | 3.34 | 0.32 | 42.65 | 0.00 |
| Gross Divorce Rate | 3.31 | 0.32 | 42.65 | 0.00 |
| Average age at divorce | 3.36 | 0.34 | 46.75 | 6.10 |
| Average age at first family planning consultation | 4.19 | 0.17 | 85.29 | 0.00 |
| **Rate of adherence to cervical cancer screening** | **4.46** | **0.17** | **91.46** | **0.00** |
| Early education and training dropout rate | 4.44 | 0.21 | 85.19 | 1.20 |
| Occupancy rate for Portuguese language courses for foreigners | 3.22 | 0.34 | 41.79 | 1.50 |
| Single-parent classic households | 3.41 | 0.32 | 48.48 | 2.90 |
| **Paternity leave utilization rate** | **4.33** | **0.21** | **87.65** | **1.20** |
| *Human Rights* |  |  |  |  |
| Number of users who refuse a medical act in the area of Sexual Reproductive Health | 4.02 | 0.25 | 74.24 | 2.90 |
| Number of undocumented migrants | 4.13 | 0.28 | 75.64 | 4.90 |
| Number of sexual offenders | 4.36 | 0.23 | 88.31 | 6.10 |
| **Number of complaints for discrimination based on gender identity** | **4.53** | **0.19** | **89.87** | **3.70** |
| **Number of complaints for discrimination based on sexual orientation** | **4.51** | **0.19** | **89.87** | **3.70** |
| *Laws, policies, regulations and strategies* |  |  |  |  |
| Number of operational health assistants fluent in English (or another foreign language) | 3.71 | 0.28 | 66.18 | 0.00 |
| **Number of CLAIs (Local Support Centers for the Integration of Migrants) available to the migrant population** | **4.37** | **0.22** | **88.46** | **4.90** |
| **Percentage of government spending on health, directed at sexual and reproductive health** | **4.44** | **0.20** | **91.36** | **1.20** |
| **Good Practices** |  |  |  |  |
| Existence of sex education in a school environment with updated content and appropriate to age and different cultural profiles | 4.63 | 0.20 | 91.46 | 0.00 |
| Existence of social marketing initiatives aimed at the use of condoms or other contraceptive methods | 4.35 | 0.19 | 86.76 | 0.00 |
| Existence of procedures in health units that guarantee the informed choice in Sexual and Reproductive Health | 4.55 | 0.18 | 92.68 | 0.00 |
| Existence of evidence-based Sexual and Reproductive Health counseling services | 4.53 | 0.19 | 92.59 | 1.20 |
| Existence of health units with Sexual and Reproductive Health skills suitable for users with different social and cultural profiles | 4.46 | 0.22 | 87.80 | 0.00 |
| Promotion of training actions for health professionals focusing on the beliefs and myths about sexuality that exist in the main migrant groups | 4.54 | 0.21 | 90.12 | 1.20 |
| Existence of a national strategy to eradicate female genital mutilation | 4.55 | 0.20 | 91.25 | 2.40 |
| Implementation of campaigns against sexual violence | 4.59 | 0.20 | 87.80 | 0.00 |
| Promotion of information sessions on condom use | 4.35 | 0.20 | 83.82 | 0.00 |
| Elaboration and availability of an antenatal, intrapartum and postnatal information guide, in different languages | 4.37 | 0.23 | 87.80 | 0.00 |
| Existence of health facilities equipped with qualified personnel to provide perinatal health care | 4.57 | 0.22 | 92.41 | 3.70 |
| Ensuring that the World Health Organization's recommendations for intrapartum care are respected | 4.60 | 0.18 | 92.50 | 2.40 |
| Implementation of behavioral change programs in sexually transmitted disease surveillance / screening units | 4.42 | 0.23 | 83.33 | 4.90 |
| Monitoring of patient safety measures aimed at controlling infections in maternity hospitals | 4.36 | 0.20 | 86.25 | 2.40 |
| Ensuring universal access to contraceptive methods | 4.63 | 0.17 | 91.36 | 1.20 |
| Existence of services that universally guarantee the right to choose the number of children and the time interval between each child | 4.38 | 0.20 | 83.54 | 3.70 |
| Fostering men's participation in the discussion of issues related to Sexual and Reproductive Health | 4.41 | 0.21 | 90.12 | 1.20 |
| Communication of information promoting safe abortion | 4.39 | 0.23 | 89.02 | 0.00 |
| Development of an integrated plan to provide recognition and treatment of complications resulting from abortion | 4.30 | 0.22 | 89.87 | 3.70 |
| Existence of legislation that allows abortion | 4.60 | 0.20 | 91.36 | 1.20 |
| Guarantee of accessibility to safe abortion, pharmacologically and / or surgically | 4.58 | 0.20 | 90.12 | 1.20 |
| Implementation of a strategy to reduce the number of maternal deaths associated with abortion | 4.60 | 0.20 | 91.25 | 2.40 |
| Greater coverage of the reasons why abortion is permitted | 3.70 | 0.36 | 64.86 | 9.80 |
| Providing advice on care to be taken for a safe abortion | 4.54 | 0.19 | 88.89 | 1.20 |
| Provision of contraception after abortion, when desired | 4.60 | 0.18 | 91.46 | 0.00 |
| Health facilities, goods, information and health services related to Sexual and Reproductive Health must be accessible to all individuals and groups without discrimination and free from obstacles | 4.74 | 0.16 | 92.68 | 0.00 |
| Promotion of awareness-raising campaigns targeting the main migrant populations about the importance of early diagnosis of cervical cancer through proper screening (Papanicolaou test) | 4.54 | 0.18 | 91.46 | 0.00 |
| Provision of training actions aimed at migrant populations to improve their navigation in the health system | 4.55 | 0.18 | 89.02 | 0.00 |
| Existence of laws and regulations that guarantee full and equal access to Sexual and Reproductive Health care | 4.65 | 0.18 | 92.59 | 1.20 |
| Guarantee of equity in the time of attendance in health consultations between migrants and non-migrants | 4.58 | 0.20 | 88.89 | 1.20 |
| Ensuring that family planning decisions are made as a couple, with special attention to migrant populations | 4.21 | 0.23 | 82.35 | 0.00 |
| Ensuring that the administrative staff (operational assistants) of the health units are fluent in English | 4.15 | 0.21 | 80.60 | 1.50 |
| Promotion of training actions for health professionals focusing on the specific cultural characteristics of the main groups of migrants in their area of ​​activity | 4.51 | 0.20 | 92.59 | 1.20 |
| Ensuring that healthcare professionals at healthcare facilities are fluent in English (or another foreign language) | 4.28 | 0.18 | 85.29 | 0.00 |
| Implementation of laws that guarantee universal access to Sexual and Reproductive Health care | 4.57 | 0.21 | 90.24 | 0.00 |
| Improving access to mobile services for disseminating Sexual and Reproductive Health to populations facing geographical barriers | 4.34 | 0.24 | 84.15 | 0.00 |
| Disclosure of the existence and strengths of CLAIs (Local Support Centers for the Integration of Migrants) with migrant populations | 4.49 | 0.23 | 89.87 | 3.70 |
| Ensuring equal access to health care for undocumented migrants | 4.60 | 0.21 | 91.46 | 0.00 |
| Implementation of a national strategy for Sexual and Reproductive Health and an action plan that includes public participation (public consultation) | 4.43 | 0.20 | 88.89 | 1.20 |
